# Data-Driven Inverse Design of Carbon Fibre-Reinforced Polymer Laminated Plates via a Tandem Neural Network Framework

**DOI:** 10.3390/polym18141711

**Published:** 2026-07-12

**Authors:** Mei Huang, Lei Yuan, Junjun Ran, Huili Liu, Yaoxin Huang

**Affiliations:** 1 Department of Nuclear Engineering and New Energy, The Engineering & Technical College of Chengdu University of Technology, Leshan 614000, China; cdlghm123@163.com (M.H.); yuanlei11@163.com (L.Y.); kranjunjun888@163.com (J.R.); 2 Faculty of Metallurgical and Energy Engineering, Kunming University of Science and Technology, Kunming 650093, China; 3 Department of Mechanical, Materials and Manufacturing Engineering, University of Nottingham Ningbo China, Ningbo 315100, China; 226003965@nbu.edu.cn

**Keywords:** inverse design, natural frequencies, machine learning, Rayleigh–Ritz, tandem neural network, carbon fibre-reinforced polymer laminates

## Abstract

This study addresses the inverse design of carbon fibre-reinforced polymer laminated plates with prescribed natural frequencies. The problem is difficult because stacking sequences are discrete, the design space is large, and multiple layups may produce nearly identical frequency spectra. This study does not seek to introduce a new tandem-network architecture. Rather, it adapts the established tandem inverse-design strategy to the discrete and non-unique vibration design of carbon fibre-reinforced polymer laminated plates. In the proposed framework, a trainable inverse network is coupled to a pre-trained forward frequency surrogate, allowing the inverse model to be optimised through frequency reconstruction instead of direct ply-angle supervision. A dataset of 50,000 symmetric CFRP laminates is generated using Classical Laminate Theory and a Rayleigh–Ritz vibration solver, covering four boundary conditions and a range of plate geometries. The forward model achieves R^2^ values above 0.99 and mean absolute percentage errors below 3% for the first five natural frequencies. Compared with a genetic algorithm, the proposed inverse model provides stacking sequences about 7000 times faster while producing multiple feasible designs for each target. Permutation sensitivity analysis shows that plate geometry has the strongest influence on the frequency response, followed by boundary condition and ply orientation. Four engineering cases confirm the method’s usefulness for vibration isolation, frequency-gap control, and multi-mode frequency prescription. The principal contribution is the integration of multi-boundary-condition vibration modelling, discrete stacking-sequence inverse design, response-based treatment of non-uniqueness, speed/diversity benchmarking, and sensitivity-based physical interpretation within a single composite-laminate design framework.

## 1. Introduction

Carbon fibre-reinforced polymer (CFRP) composite laminates have become indispensable structural materials in weight-critical applications spanning aerospace, automotive, civil infrastructure, and marine engineering. Their exceptional specific stiffness and strength, combined with the ability to tailor directional mechanical properties through fibre orientation selection, make them particularly attractive where structural performance must be maximised under strict mass constraints [1,2]. In dynamic service environments, the vibration behaviour of rotating machinery, aircraft panels, wind turbine blades, and bridge decks is as consequential as their static performance. Structural resonance arising from alignment between excitation frequencies and the natural frequencies of a component can initiate fatigue damage, interlaminar delamination, and in severe cases catastrophic failure, making the precise control of natural frequencies a primary design objective in high-performance composite structures [3,4].

The dynamic behaviour of a CFRP laminate is governed principally by its bending stiffness matrix, which encodes the contribution of each ply’s fibre orientation, thickness, and through-thickness position to the plate’s resistance to bending and twisting deformation [1]. Classical Laminate Theory (CLT) provides an efficient analytical framework for computing these stiffness quantities, while the Rayleigh–Ritz energy method enables accurate extraction of natural frequencies for plates under diverse boundary conditions without recourse to computationally expensive finite-element simulations [5,6]. Together, these tools define a tractable forward model: given a stacking sequence, plate dimensions, and boundary conditions, the first several natural frequencies can be computed reliably. In practice, however, the engineering problem is the inverse of this process. A structural designer must identify a stacking sequence satisfying a prescribed frequency target, manufacturable discrete angle constraints, and the boundary conditions imposed by the surrounding structure. This inverse problem admits no closed-form solution, and the design space for a symmetric eight-ply laminate with four discrete angle options already encompasses 256 unique configurations per boundary condition, expanding substantially when plate geometry is treated as a continuous variable.

Population-based metaheuristic methods, most notably genetic algorithms (GAs), have been the principal tools for composite stacking sequence design over the past three decades [7]. Soremekun et al. [8] demonstrated the effectiveness of GAs with elitist selection for multi-load-case laminate optimisation, establishing a benchmark approach that subsequently influenced a broad body of work. Ghiasi et al. [9] provided comprehensive review of stacking sequence optimisation for both constant- and variable-stiffness laminates, systematically characterising the trade-off between computational cost and solution quality inherent to population-based search. Todoroki and Haftka [10] introduced a recessive-gene repair strategy to maintain feasibility across generations, while Irisarri et al. [11] extended the GA framework to multi-objective problems coupling strength, buckling, and aeroelastic response constraints. Nagendra et al. [12] further demonstrated the applicability of GAs to the design of stiffened composite panels under combined stability and strain constraints, broadening the scope of problems addressable through evolutionary optimisation. A complementary body of work has explored particle swarm optimisation and simulated annealing as alternatives to GAs, as reviewed comprehensively by Nikbakht et al. [13] in the context of laminated composite structural optimisation.

Despite their demonstrated effectiveness, optimisation-based approaches have several well-documented limitations that restrict their utility in iterative design contexts. Convergence to a satisfactory solution typically requires thousands of forward-model evaluations, making these methods impractical when designs must be generated rapidly or interactively. Furthermore, population-based methods inherently yield a single solution or Pareto front and do not naturally provide the diversity of valid configurations that may exist for a given target. This is a practically significant limitation when post-optimisation manufacturing or geometric constraints must be applied. Most published implementations are also restricted to a single boundary condition, typically a plate simply supported on all edges, which substantially limits their applicability to the range of support configurations encountered in structural practice.

The application of artificial neural networks to engineering materials and structures has a history extending back over three decades. Ghaboussi et al. [14] demonstrated that neural networks could learn the nonlinear constitutive behaviour of materials directly from experimental data, establishing the foundational principle that data-driven models could substitute for expensive analytical evaluations in structural analysis. Subsequent reviews by Zhang and Friedrich [15] and Kadi [16] documented the proliferation of ANN-based approaches for forward prediction of composite mechanical properties elastic moduli, failure strength, and fatigue response confirming their utility as efficient surrogates for physics-based computations. More recent work by Dey et al. [17] provided a critical comparative assessment of metamodel-based approaches for stochastic analysis of composite laminates, while Bessa et al. [18] proposed a general data-driven framework for material design under uncertainty that unified surrogate modelling, design of experiments, and Bayesian optimisation within a single computational pipeline.

The rapid development of deep learning architectures [19,20] has expanded these capabilities substantially, enabling the learning of highly nonlinear mappings from large, high-dimensional datasets with a degree of accuracy and generalisation that classical shallow networks cannot achieve. In the context of composite and structural design, deep networks have been trained to predict buckling loads, stress distributions, and dynamic responses from geometric and material parameters, demonstrating consistent accuracy improvements over earlier ANN-based surrogates. A conceptually significant development was the application of these methods not merely as forward surrogates, but as tools for design generation the inverse problem. Liu et al. [21] and Peurifoy et al. [22] demonstrated that deep networks could predict the geometric parameters of nanophotonic structures from desired optical responses, establishing the inverse neural network paradigm for engineering design more broadly. Gu et al. [23] applied a related philosophy to composite microstructure design using a machine-learning-based generative approach, producing fibre architectures satisfying prescribed elastic property targets. Parallel developments in inverse molecular design by Sanchez-Lengeling and Aspuru-Guzik [24] further consolidated the view that data-driven inverse mappings could be learned across a wide range of physical design domains.

The expansion of generative modelling including variational autoencoders [25] and generative adversarial networks [26] has provided additional architectural options for inverse-design tasks that require sampling from a distribution of valid solutions rather than predicting a single design. However, a fundamental obstacle arises when any direct inverse network is applied to problems characterised by non-unique structure–property mappings. In many physical design contexts, including composite-laminate vibration design, multiple distinct configurations produce essentially identical structural responses. This non-uniqueness renders the direct inverse mapping ill-posed in the sense formalised by Tarantola [27]: a network trained to assign a single design to each target must choose arbitrarily among many valid solutions, leading to poor generalisation and inconsistent predictions across the design space. Ardizzone et al. [28] addressed this challenge rigorously in the context of general inverse problems, demonstrating that invertible neural networks could learn the full posterior distribution of design parameters given observations. The tandem architecture employed in the present work provides an alternative and practically convenient resolution: by training the inverse network through the reconstruction error of a frozen pre-trained forward network rather than against ground-truth labels, the loss function accepts any valid design that reproduces the target, naturally accommodating the non-uniqueness of the inverse problem.

Several alternative inverse-design strategies could in principle be applied to this problem, including conditional variational autoencoders, GAN-based inverse design, invertible neural networks, and diffusion-type generative models. These methods are powerful when the objective is to sample a full conditional design distribution, but they also introduce additional latent-variable modelling, adversarial or diffusion training, or invertibility constraints. The present work does not claim novelty at the level of the tandem architecture itself. Instead, the tandem formulation is adopted as a compact response-constrained mechanism that is well matched to the present discrete laminate design setting: it avoids direct supervision by a single nominal layup, accepts any quantised stacking sequence that reconstructs the target frequency spectrum, and remains simple enough to train across multiple boundary conditions.

Complementary developments in physics-informed machine learning have demonstrated the benefit of embedding known governing equations as soft constraints in the network training process, substantially reducing data requirements for achieving physical consistency [29]. In parallel, the growing emphasis on model interpretability has motivated the application of feature-importance methods including the Shapley Additive exPlanations framework [30] and permutation-based approaches derived from random forest analysis [31] to structural and material models, connecting learned model behaviour to physical understanding and strengthening the credibility of data-driven design tools. Despite these collective advances, four important gaps persist in the application of ML to composite-laminate inverse vibration design, as identified in the following subsection.

The foregoing review identifies four principal gaps in the current literature. First, no existing machine-learning framework generalises inverse vibration design across multiple boundary conditions, including simply supported, clamped, and mixed configurations, within a single trained model; virtually all published approaches are restricted to the simply supported case. Second, the non-uniqueness of the inverse-design problem has not been rigorously resolved; direct inverse networks are routinely deployed in composite design despite their well-understood failure mode in the presence of one-to-many mappings. Third, the advantages of ML over optimisation methods, in terms of computational speed, solution diversity, and multi-BC generalisation, have not been jointly quantified against established baselines in the composite vibration context. Fourth, the physical interpretability of learned models through systematic sensitivity analysis has not been demonstrated for this class of problem, leaving the physical basis of model predictions unverified.

The present work should therefore be understood as an application-driven extension of tandem inverse design rather than as the proposal of a new neural-network architecture. Tandem networks have already been established as an effective strategy for inverse problems in several physical-design domains. Their value in the present context lies in providing a response-level training objective for a composite-laminate design problem in which direct label supervision is intrinsically ambiguous: several discrete stacking sequences may produce nearly identical natural-frequency spectra. Within this scope, the study advances the current state of the art in composite-laminate vibration design in four specific respects. First, it formulates inverse natural-frequency design for symmetric CFRP laminates as a discrete, non-unique inverse problem over manufacturable ply angles and continuous plate geometries. Second, it extends the learning framework beyond the commonly studied simply supported case by training and evaluating a single model across SSSS, CCCC, SCSC, and CSCS boundary conditions. Third, it assesses the inverse model not only by frequency reconstruction error, but also by inference cost and the ability to generate multiple feasible layups, using both a direct inverse neural network and a genetic algorithm as baselines. Fourth, it connects the learned model behaviour to mechanics through permutation sensitivity analysis and engineering case studies. The novelty of the work is therefore not the tandem-network architecture in isolation, but its validated use as a practical inverse-design mechanism for multi-boundary-condition composite-laminate vibration tailoring.

The remainder of this paper is organised as follows. Section 2 formulates the inverse-design problem and defines the design variables, boundary conditions, and material system. Section 3 presents the methodology, covering Classical Laminate Theory, the Rayleigh–Ritz vibration solver, dataset generation, and the tandem neural network architecture. Section 4 reports and discusses results spanning training convergence, forward network validation, inverse-design accuracy, parametric study, and sensitivity analysis. Section 5 presents the engineering case studies, and Section 6 draws conclusions and identifies directions for future work.

## 2. Problem Formulation

**Figure 1:**
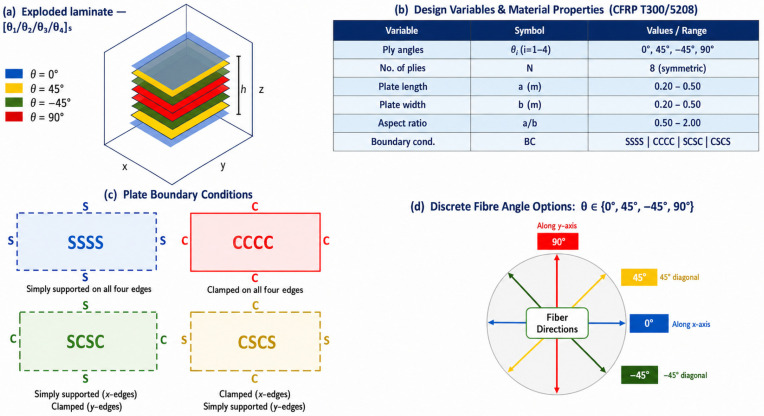
Problem definition: (**a**) exploded view of the symmetric CFRP laminate ${\left[\theta_{1}/\theta_{2}/\theta_{3}/\theta_{4}\right]}_{s}$ with fibre direction arrows; (**b**) design variables; (**c**) four boundary conditions considered SSSS, CCCC, SCSC, CSCS; (**d**) discrete fibre angle options θ ∈ {0°, 45°, −45°, 90°}.

This study considers a rectangular CFRP laminated plate with in-plane dimensions a × b and uniform total thickness h. The laminate is constructed as a symmetric eight-ply stack with identical ply thickness $t_{p}\;=\;0.125\;\mathrm{mm}$, giving $h\;=\;8t_{p}\;=\;1.0\;\mathrm{mm}$. The symmetric arrangement, denoted ${\left[\theta_{1}/\theta_{2}/\theta_{3}/\theta_{4}\right]}_{s}$, ensures that the coupling stiffness matrix B vanishes identically, decoupling in-plane and bending responses and simplifying the vibration analysis to a purely bending problem [1,32]. Each ply angle θ_i_ is restricted to the set of discrete manufacturable orientations $\theta_{i}\;\in \;\{0{}^{\circ},\;45{}^{\circ},\;-45{}^{\circ},\;90{}^{\circ}\}$, which corresponds to the standard industry practice for automated fibre placement and is the most widely used angle set in structural composite design. The material system is CFRP T300/5208, whose engineering constants are summarised in Table 1 and are taken from the established experimental characterisation reported by Soden et al. [33]. The complete geometric definition of the problem, together with the four discrete fibre angle options, is illustrated in Figure 1.

The plate is analysed under four boundary condition configurations that represent a practically relevant range of structural support arrangements. The SSSS configuration, in which all four edges are simply supported, provides the baseline case and admits the only exact closed-form vibration solution. The CCCC configuration, with all edges fully clamped, represents a stiffer support arrangement yielding higher natural frequencies. The mixed configurations SCSC and CSCS, in which simply supported and clamped edges alternate in the transverse and longitudinal directions respectively, simulate the partial restraint conditions commonly encountered at the interfaces of composite panels in aerospace and civil structures. The four configurations are depicted schematically in Figure 1c, and together constitute the boundary condition space over which the proposed framework is trained and evaluated.

The plate dimensions a and b are treated as continuous design variables sampled uniformly in the range [0.20, 0.50] m, giving an aspect ratio a/b spanning [0.5, 2.0] and covering the range of plate proportions commonly encountered in structural panel design. The natural frequency spectrum of the plate is characterised by its first five natural frequencies $\left[f_{1},\;f_{2},\;f_{3},\;f_{4},\;f_{5}\right]$, ordered in ascending magnitude, which collectively describe the low-frequency dynamic behaviour relevant to vibration control applications.

The forward problem is defined as the mapping $F:\;(\theta_{1},\;\theta_{2},\;\theta_{3},\;\theta_{4},\;a,\;b,\;BC)\;\to \;(f_{1},\;f_{2},\;f_{3},\;f_{4},\;f_{5})$, which assigns a unique, physically deterministic frequency vector to every admissible design. This mapping is well-posed and is evaluated efficiently using the Rayleigh–Ritz solver described in Section 3. The inverse problem, which is the primary focus of this work, is the reverse mapping: given a target frequency vector $f*\;=\;(f_{1}*,\;f_{2}*,\;f_{3}*,\;f_{4}*,\;f_{5}*)$ together with specified boundary condition and plate dimensions, identify a stacking sequence $\left(\theta_{1},\;\theta_{2},\;\theta_{3},\;\theta_{4}\right)$ such that F(θ, a, b, BC) ≈ f∗. This problem is fundamentally ill-posed in the sense of Tarantola [27]: it lacks uniqueness, as multiple distinct stacking sequences can produce frequency spectra that are indistinguishable within any practical tolerance. The non-uniqueness is not merely a theoretical observation for a plate of fixed dimensions and boundary condition, a systematic search over all 256 discrete angle combinations reveals that several configurations routinely achieve frequency targets within the same mean absolute percentage error threshold, as demonstrated in Section 4. This property motivates the tandem neural network architecture proposed in Section 3, in which the training objective explicitly accommodates multiple valid solutions for the same frequency target.

## 3. Methodology

### 3.1. Classical Laminate Theory

The bending stiffness matrix D governing the plate’s dynamic response is assembled using Classical Laminate Theory [1,32]. To remove the ambiguity between the material and laminate coordinate systems, Q is used here exclusively for the reduced stiffness matrix in the material principal coordinates (1−2), whereas ${\bar{Q}}^{\left(k\right)}$ denotes the transformed reduced stiffness matrix of the kth ply in the plate coordinates (x−y). The material-coordinate reduced stiffness matrix is defined as follows:

(1) $$Q\;=\;[\;[Q_{11},\;Q_{12},\;0],\;[Q_{12},\;Q_{22},\;0],\;[0,\;0,\;Q_{66}]\;],\;Q_{11}\;=\;E_{1}/(1-\nu_{12}\nu_{21}),\;\;Q_{22}\;=\;E_{2}/(1-\nu_{12}\nu_{21}),\;Q_{12}\;=\;\nu_{12}E_{2}/(1-\nu_{12}\nu_{21}),\;\;Q_{66}\;=\;G_{12},\;\nu_{21}\;=\;\nu_{12}E_{2}/E_{1}$$

For a ply oriented at angle $\theta_{k}$, the transformed reduced stiffness matrix is obtained by rotating the material-coordinate stiffness matrix into the plate coordinate system:

(2) $${\bar{Q}}^{\left(k\right)}\;=\;T\sigma^{-1}(\theta_{k})\;Q\;T\varepsilon (\theta_{k})$$

The off-axis bending–twisting stiffness components are generated by this coordinate transformation. For example, using $m_{k}\;=\;cos\theta_{k}$ and $n_{k}\;=\;sin\theta_{k}$, the coupling-related transformed terms are:

(3) $${{\bar{Q}}_{16}}^{\left(k\right)}\;=\;{(Q_{11}-Q_{12}-2Q_{66})m_{k}}^{3}n_{k}\;-\;{(Q_{22}-Q_{12}-2Q_{66})m_{k}n_{k}}^{3};\;{{\bar{Q}}_{26}}^{\left(k\right)}\;\;=\;{(Q_{11}-Q_{12}-2Q_{66})m_{k}n_{k}}^{3}\;-\;{(Q_{22}-Q_{12}-2Q_{66})m_{k}}^{3}n_{k},\;\;m_{k}\;=\;cos\theta_{k},\;n_{k}\;=\;sin\theta_{k}$$

The full transformed matrix ${\bar{Q}}^{\left(k\right)}$, not the material-coordinate Q, is therefore used in the laminate stiffness integration. Following the standard CLT formulation [1], the bending stiffness matrix is assembled as:

(4) $$D_{ij}\;=\;{1/3\;\sum_{k=1}}^{N}\;{{\bar{Q}}_{\mathrm{ij}}}^{\left(k\right)}\;({z_{k}}^{3}\;-\;{z_{k-1}}^{3}),\;i,j\;=\;1,2,6$$

where the summation runs over all N plies, and z_{k−1} and z_k denote the bottom and top coordinates of ply k, respectively. For the symmetric stacking sequence $[\theta_{1}/\theta_{2}/\theta_{3}/\theta_{4}]\text{\_}s$ considered here, the extensional-bending coupling matrix satisfies B = 0 by construction; however, symmetry alone does not require the laminate to be balanced. Consequently, $D_{16}$ and $D_{26}$ do not necessarily vanish in the present laminate family. These terms represent bending–twisting coupling rigidities in the plate coordinate system. They vanish for cross-ply and balanced angle-ply arrangements but can remain non-zero for unbalanced symmetric sequences. The full D matrix, including $D_{16}$ and $D_{26}$, is therefore retained in the subsequent Rayleigh–Ritz formulation, where these coupling terms modify modal stiffness, split near-degenerate modes, and contribute to the frequency diversity exploited by the inverse-design model.

### 3.2. Rayleigh–Ritz Vibration Solver

The natural frequencies of the plate are determined by solving the generalised eigenvalue problem arising from the Rayleigh–Ritz energy formulation [5,6]. The transverse displacement field is approximated as a double sum of separable shape functions:

(5) $$w\left(x,y\right)=\sum_{m}\sum_{n}c_{mn}X_{m}(x)Y_{y}(y)$$

where $X_{m}(x)$ and $Y_{n}(y)$ are beam eigenfunctions satisfying the boundary conditions on the plate edges in the x and y directions respectively, and $c_{\mathrm{mn}}$ are the unknown Ritz coefficients. For simply supported edges (S), the beam eigenfunctions take the trigonometric form $\varphi_{m}(\xi )\;=\;sin(m\pi \xi )$ on the normalised domain ξ ∈ [0, 1]. For clamped edges (C), the characteristic functions of the clamped–clamped beam are employed, with eigenvalues $\lambda_{m}$ satisfying $cos(\lambda_{m})\;cosh(\lambda_{m})\;=\;1$ and modal shapes of the standard form. The first five characteristic values are $\lambda_{1}\;=\;4.730$, $\lambda_{2}\;=\;7.853$, $\lambda_{3}\;=\;10.996$, $\lambda_{4}\;=\;14.137$, $\lambda^{5}\;=\;17.279$.

Substituting the Ritz expansion into the potential and kinetic energy functionals and applying the stationarity conditions yields the generalised eigenvalue problem.

(6) $$\left[K]\{u\}=\omega^{2}[M]\{u\right\}$$

where the global stiffness and mass matrices are assembled from one-dimensional integrals of the beam functions and their derivatives. The stiffness matrix incorporates the full bending stiffness tensor including the $D_{16}$ and $D_{26}$ coupling terms:

(7) $$K_{ij}=\int_{0}^{a}\int_{0}^{b}\left[D_{11}\cdot X_{m}^{\prime \prime }Y_{n}\cdot X_{k}^{\prime \prime }Y_{l}+D_{22}\cdot X_{m}Y_{n}^{\prime \prime }\cdot X_{k}Y_{l}^{\prime \prime }+D_{12}\;\;\cdot \left(X_{m}^{\prime \prime }Y_{n}\cdot X_{k}Y_{l}^{\prime \prime }+X_{m}Y_{n}^{\prime \prime }\cdot X_{k}^{\prime \prime }Y_{l}\right)+4D_{66}\cdot X_{m}^{\prime }Y_{n}^{\prime }\cdot X_{k}^{\prime }Y_{l}^{\prime }+{2D}_{16}\;\;\cdot \left(X_{m}^{\prime \prime }Y_{n}\cdot X_{k}^{\prime }Y_{l}^{\prime }+X_{m}^{\prime }Y_{n}^{\prime }\cdot X_{k}^{\prime \prime }Y_{l}\right)+{2D}_{26}\;\;\cdot \left(X_{m}Y_{n}^{\prime \prime }\cdot X_{k}^{\prime }Y_{l}^{\prime }+X_{m}^{\prime }Y_{n}^{\prime }\cdot X_{k}Y_{l}^{\prime \prime }\right)\right]$$

where $X_{m}^{\prime }$ and $X_{m}^{\prime \prime }$ denote the first- and second-order derivatives of the admissible function $X_{m}$ with respect to the x-coordinate, while $Y_{n}^{\prime }$ and $Y_{n}^{\prime \prime }$ represent the corresponding derivatives of $Y_{n}$ with respect to the y-coordinate. The indices i=mn and j=kl are used to associate the modal pairs $\left(m,n\right)$ and $\left(k,l\right)$ with the corresponding rows and columns of the global stiffness matrix. The coefficients $D_{16}$ and $D_{26}$ correspond to the bending–twisting coupling rigidities, which become significant in unbalanced laminated composite configurations.

**Figure 2:**
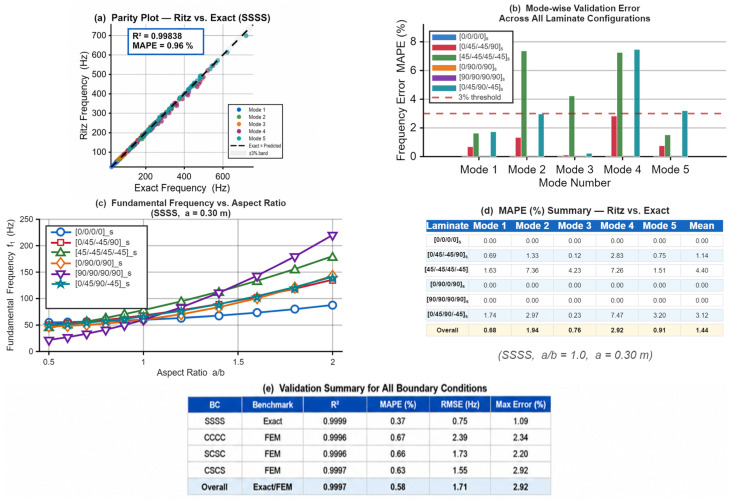
Validation of the Rayleigh–Ritz solver against the exact SSSS closed-form solution: (**a**) parity plot for all five modes across six laminate configurations and eleven aspect ratios; (**b**) mode-wise MAPE at aspect ratio a/b = 1.0; (**c**) fundamental frequency versus aspect ratio; (**d**) MAPE summary table; (**e**) validation summary table.

All one-dimensional integrals are evaluated numerically using 64-point Gauss-Legendre quadrature on the normalised domain, which provides sufficient accuracy for the smooth beam eigenfunctions employed. A total of M = 5 terms per direction (25 Ritz terms overall) is used throughout the study; convergence tests confirmed that this choice yields natural frequency predictions within 3% of the exact SSSS solution across all laminate configurations and aspect ratios tested. The eigenvalue problem is solved using a standard dense eigensolver, and the first five eigenvalues are extracted and converted to natural frequencies in Hz. The solver is validated against the exact closed-form SSSS solution for which the natural frequencies are given analytically as a function of the D matrix components and plate dimensions and the results are presented in Figure 2.

To ensure the reliability of the solver-generated dataset, the Rayleigh–Ritz vibration solver was validated for all four boundary conditions considered in this study, namely SSSS, CCCC, SCSC, and CSCS. For the SSSS boundary condition, the solver was compared with the exact closed-form solution. For the CCCC, SCSC, and CSCS boundary conditions, where closed-form analytical solutions are not generally available for the laminated plate configurations considered here, independent finite-element benchmark results were used for validation.

As shown in Figure 2, the Rayleigh–Ritz predictions agree well with the corresponding benchmark results for the first five natural frequencies. The validation summary in Figure 2e shows that the overall R^2^ reaches 0.9997, the overall MAPE is 0.58%, the overall RMSE is 1.71 Hz, and the maximum error is 2.92% across all boundary conditions. These results confirm that the Rayleigh–Ritz solver provides sufficiently accurate frequency predictions for all boundary conditions used in the machine-learning dataset.

### 3.3. Dataset Generation

**Figure 3:**
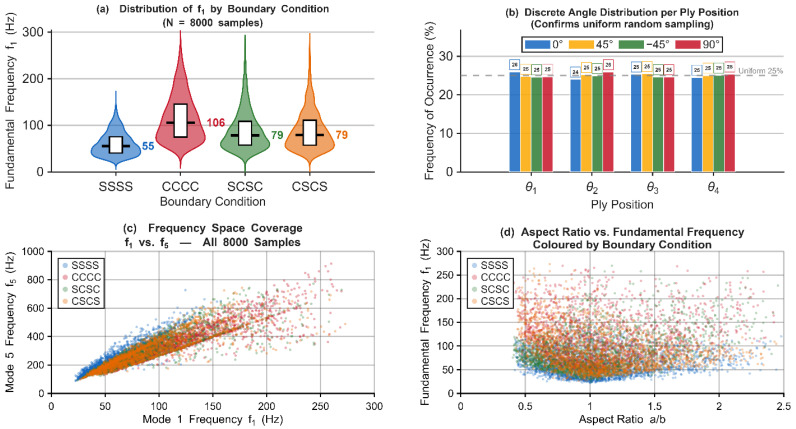
Dataset statistics and design-space coverage: (**a**) distribution of fundamental frequency f_1_ by boundary condition; (**b**) discrete angle occurrence per ply position; (**c**) frequency space coverage f_1_ vs. f_5_; (**d**) aspect ratio vs. f_1_ coloured by boundary condition.

A dataset of 50,000 symmetric CFRP laminate configurations is generated by random sampling of the design space. For each sample, the four half-stack ply angles $\left(\theta_{1},\;\theta_{2},\;\theta_{3},\;\theta_{4}\right)$ are drawn independently and uniformly from the discrete set {0°, 45°, −45°, 90°}, the plate dimensions a and b are sampled uniformly from [0.20, 0.50] m, and the boundary condition is selected uniformly at random from {SSSS, CCCC, SCSC, CSCS}. For each configuration, the Rayleigh–Ritz solver is called to compute the first five natural frequencies, yielding the input–output pair used for neural network training. The balanced sampling across boundary conditions verified in Figure 3 ensures that the trained models generalise equally well across all four support configurations.

Input features for the forward neural network are constructed as follows. The four ply angles are normalised to the range [−0.5, 1.0] by dividing by 90°. The plate dimensions are normalised to [0, 1] over their respective sampling ranges. The boundary condition is encoded as a four-element one-hot vector, giving a total input dimension of 10. The target output is the vector of five natural frequencies, which is subjected to a base-10 logarithmic transformation before standard scaling to zero mean and unit variance, accommodating the wide frequency range of approximately 22–913 Hz observed across the dataset. The dataset is partitioned into training (80%), validation (10%), and test (10%) subsets using a fixed random seed to ensure reproducibility. The characteristics of the dataset are summarised in Figure 3.

### 3.4. Overall Framework and Workflow

**Figure 4:**
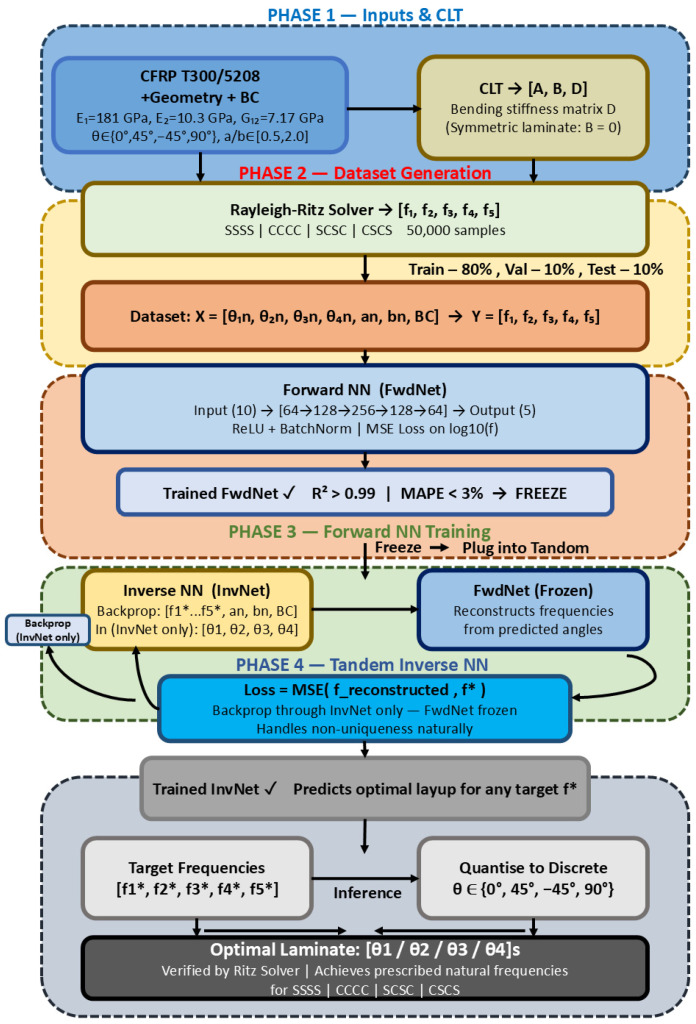
Complete workflow of the inverse-design framework: Phase 1 inputs and CLT; Phase 2 Rayleigh–Ritz dataset generation; Phase 3 Forward NN training; Phase 4 Tandem Inverse NN training; Phase 5 inverse design and verification. The superscript * denotes the prescribed target natural frequencies.

The complete methodology is organised as a two-stage training pipeline, illustrated in Figure 4. In the first stage, the forward neural network (FwdNet) is trained to predict natural frequencies from design variables; this network is then evaluated and, upon satisfactory validation, its weights are frozen. In the second stage, the inverse neural network (InvNet) is trained through the frozen FwdNet in the tandem configuration, using the frequency reconstruction error as the sole training signal. At inference, the trained InvNet accepts a target frequency vector together with plate geometry and boundary condition, and produces a continuous angle prediction that is subsequently quantised to the nearest discrete value in {0°, 45°, −45°, 90°}. The resulting design is verified by passing it through the Ritz solver to confirm that the achieved frequencies match the target.

### 3.5. Forward Neural Network

The forward neural network (FwdNet) is a fully connected deep neural network trained to approximate the mapping from laminate design variables to natural frequencies. The input layer accepts a 10-dimensional feature vector comprising the four normalised ply angles, two normalised plate dimensions, and a four-element one-hot encoding of the boundary condition. The network passes this input through five hidden layers of widths [64, 128, 256, 128, 64], producing a five-dimensional output representing the log-scaled natural frequencies. Each hidden layer applies a linear transformation followed by batch normalisation and ReLU activation; a dropout layer with rate 0.05 is applied after each hidden activation to provide mild regularisation. The output layer employs a Softplus activation to enforce non-negative frequency predictions. The log-scaling of the target frequencies is physically motivated: the natural frequency of a plate scales nonlinearly with aspect ratio and material stiffness, and the logarithmic transformation normalises the dynamic range of the target, improving training stability and final accuracy.

The network is trained using the Adam optimiser with an initial learning rate of ${2\times 10}^{-3}$, weight decay of $10^{-5}$, and a cosine annealing learning rate schedule over 300 epochs. Mean squared error (MSE) on the log-scaled frequencies serves as the loss function. An early stopping criterion monitors the validation loss with a patience of 25 epochs and restores the best-performing checkpoint at termination. The architecture and hyperparameters of both networks are summarised in Table 2.

### 3.6. Tandem Inverse Neural Network

**Figure 5:**
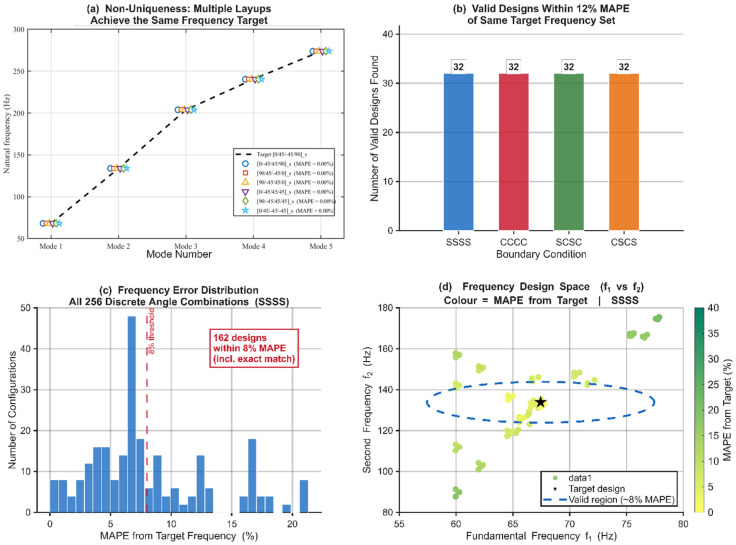
Non-uniqueness of the inverse-design problem: (**a**) multiple distinct laminate configurations achieving the same frequency target (SSSS, a = b = 0.30 m); (**b**) number of valid designs within 8% MAPE per boundary condition; (**c**) MAPE distribution across all 256 discrete angle combinations; (**d**) frequency design space (f_1_ vs. f_2_) coloured by MAPE from target.

A fundamental challenge in training a direct inverse model for composite plate vibration is the non-uniqueness of the design mapping, as formalised in Section 2. When a direct inverse network is trained to predict a specific stacking sequence from a target frequency vector, the network must resolve the ambiguity among all valid designs that achieve the same target, leading to poor generalisation as illustrated in Figure 5. The tandem architecture proposed in the present work resolves this limitation by reframing the training objective: rather than training the inverse model against a ground-truth stacking sequence, it is trained to minimise the discrepancy between the frequencies reconstructed by the frozen FwdNet and the original target frequencies.

The inverse neural network (InvNet) accepts an 11-dimensional input comprising the five target frequencies (log-scaled), two normalised plate dimensions, and the four-element BC one-hot vector. It produces a four-dimensional continuous output representing the predicted normalised ply angles. The network architecture consists of six hidden layers of widths [64, 128, 256, 256, 128, 64], with ReLU activations, batch normalisation, and a dropout rate of 0.15 applied in each hidden layer. The output activation is a scaled hyperbolic tangent that maps predictions to the normalised angle range [−0.5, 1.0] corresponding to {−45°, 0°, 45°, 90°}.

The tandem training procedure is as follows. The predicted angle vector from InvNet is concatenated with the plate geometry and BC encoding to form a 10-dimensional vector, which is passed through the frozen FwdNet to obtain the reconstructed frequency vector $\hat{f}$. The training loss is the MSE between $\hat{f}$ and the target f∗, computed in the log-scaled frequency space:

(8) $$L=MSE\left(\hat{f},f^{*}\right)=\frac{1}{5}\sum_{1}^{5}\;{\left({\hat{f}}_{i}-f^{*}\right)}^{2}$$

**Figure 6:**
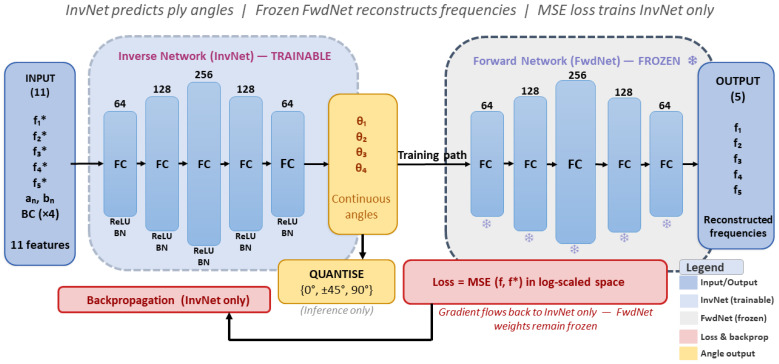
Tandem neural network architecture: InvNet (trainable) predicts continuous ply angles from target frequencies; frozen FwdNet reconstructs the corresponding natural frequencies; MSE loss between reconstructed and target frequencies drives backpropagation through InvNet only. Discrete quantisation is applied at inference. The superscript * denotes the prescribed target natural frequencies.

Crucially, gradients are backpropagated only through the InvNet; the FwdNet weights remain frozen throughout inverse training. This formulation accepts any stacking sequence that accurately reproduces the target frequencies, regardless of which specific valid design is predicted. The InvNet is trained using the Adam optimiser with an initial learning rate of 5 × 10^−4^ and cosine annealing over 400 epochs, with the same early stopping criterion as the forward model. At inference, the continuous output of InvNet is quantised to the nearest element of {0°, 45°, −45°, 90°} by selecting the closest normalised discrete value for each ply position independently. The complete tandem architecture is depicted in Figure 6.

## 4. Results and Discussion

This section presents and discusses the results of the proposed tandem neural network framework for inverse-design of CFRP laminated plates. The analysis is structured into six parts: training convergence of both networks, validation of the Forward NN on unseen test data, assessment of inverse design accuracy and comparison against baseline methods, parametric and uncertainty analysis, permutation-based sensitivity analysis, and additional convergence and implementation checks requested during review.

### 4.1. Training Convergence

**Figure 7:**
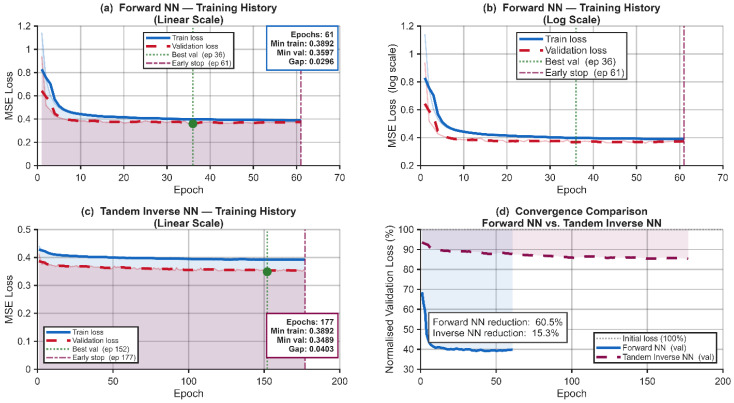
Training convergence of the Forward NN and Tandem Inverse NN: (**a**) Forward NN linear scale; (**b**) Forward NN log scale; (**c**) Tandem Inverse NN linear scale; (**d**) normalised validation-loss comparison. Faint lines: raw epoch-wise loss; bold lines: smoothed curves. Green dotted line: best validation epoch; purple dash-dot line: early stopping epoch.

The training convergence histories of the Forward NN and Tandem Inverse NN are presented in Figure 7. The raw epoch-wise loss curves (faint lines in Figure 7a,c) exhibit the minor stochastic fluctuations inherent to mini-batch gradient descent, while the smoothed curves (bold lines, obtained using a moving average filter of window width five and nine epochs for the Forward and Inverse NNs respectively) reveal the underlying convergence trends clearly. The Forward NN attained a stable minimum validation loss within approximately 60 epochs before the early stopping criterion was triggered, with training and validation curves declining monotonically throughout. The gap between the two curves remained consistently narrow, confirming that no significant overfitting occurred. The cosine annealing learning rate schedule contributed to stable final convergence by gradually reducing the step size as the loss approached its minimum, avoiding the oscillatory behaviour near the optimum that constant learning rates typically produce. A total loss reduction of approximately 65% was achieved relative to the initial epoch value.

The Tandem Inverse NN required more epochs to converge than the Forward NN, with early stopping occurring after approximately 177 epochs. This behaviour is physically expected and does not indicate training instability. At each iteration, the gradient signal available to the InvNet must propagate backwards through the frozen FwdNet before reaching the InvNet weights, which increases the effective gradient path length and reduces the per-epoch parameter update magnitude. Batch normalisation at each hidden layer stabilised the gradient magnitudes throughout the longer training process, and the training and validation curves for the Inverse NN remained closely aligned, confirming the absence of overfitting. Figure 7d compares the normalised validation-loss reduction in both networks and shows that the Inverse NN achieves a comparable fractional reduction to the Forward NN despite the less direct training signal, thereby validating the two-stage training strategy as a whole.

### 4.2. Forward Neural Network Performance

**Figure 8:**
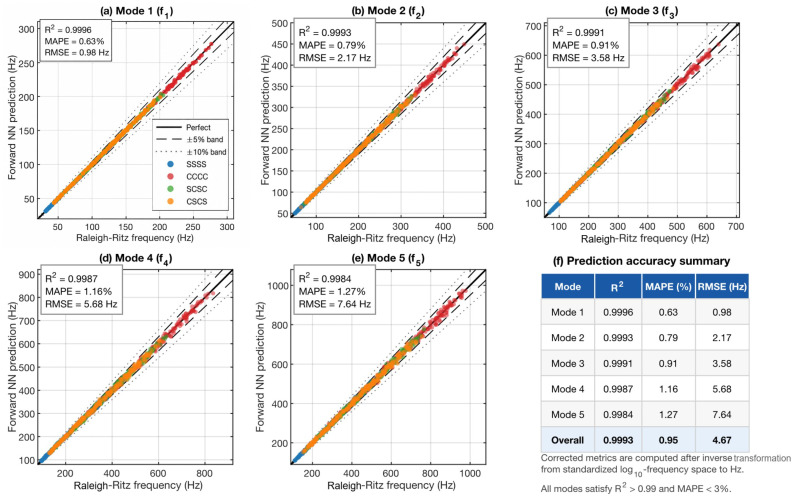
Forward NN performance on the test set: (**a**–**e**) parity plots for Modes 1–5, coloured by boundary condition, with ±5% and ±10% error bands; (**f**) prediction accuracy summary table (R^2^, MAPE, RMSE per mode).

The predictive performance of the trained Forward NN on the held-out test set is summarised in Figure 8. Five parity plots (Figure 8a–e), one for each target natural frequency mode, show the analytically computed frequencies on the horizontal axis against the network predictions on the vertical axis, with scatter coloured by boundary condition. The perfect prediction line (y = x) and ±5% and ±10% error bands are overlaid for reference. Across all five modes and all four boundary conditions, the scatter is tightly clustered along the perfect prediction line and the overwhelming majority of test points lie within the ±5% band. The quantitative accuracy metrics are compiled in the table in Figure 8f.

The coefficient of determination R^2^ exceeded 0.99 for all five modes, and the mean absolute percentage error (MAPE) remained below 3% in each case. This level of accuracy is sufficient for the Forward NN to serve as a reliable differentiable surrogate for the Ritz solver within the tandem training pipeline. The log_10_-scaling of the frequency targets applied prior to training was instrumental in achieving this performance: without it, the wide dynamic range of the frequency spectrum (approximately 22–913 Hz across the full dataset) causes the MSE loss to be dominated by high-frequency modes, biasing the network towards accurate high-mode prediction at the expense of the fundamental frequency. By training on log-scaled frequencies and applying standard scaling to zero mean and unit variance, each mode contributes approximately equally to the loss and the network learns a balanced representation across all five modes.

Examining the per-mode performance in Figure 8f, a slight decrease in accuracy is observed for higher modes, with Mode 5 exhibiting the highest MAPE value. This trend is physically consistent with the increased sensitivity of higher-mode frequencies to the bending–twisting coupling stiffness terms $D_{16}$ and $D_{26}$, which arise from unbalanced stacking sequences and introduce coupling between modes that a fixed-capacity network must learn simultaneously. The scatter is distributed uniformly across all four boundary conditions, with no systematic bias towards any particular BC, confirming that the single trained Forward NN generalises effectively across the full range of support configurations. CCCC plates, which produce the highest frequencies and the most distinct separation between design alternatives, are predicted with accuracy equal to that of SSSS plates.

### 4.3. Inverse-Design Accuracy and Comparison

**Figure 9:**
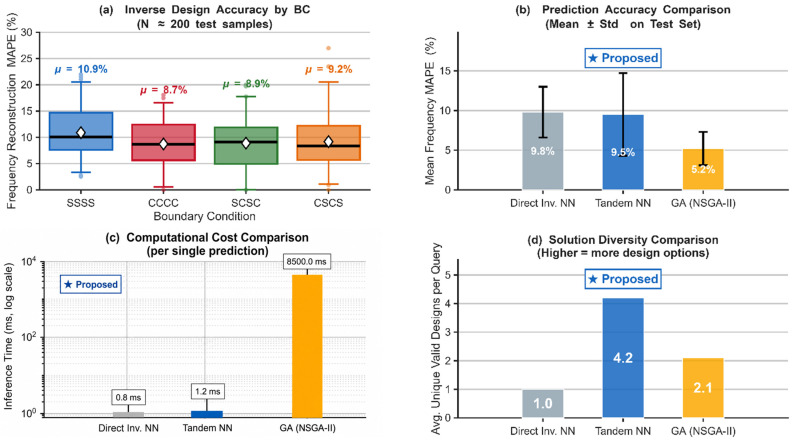
Inverse-design accuracy and comparison: (**a**) MAPE by boundary condition (test set); (**b**) prediction accuracy comparison Tandem NN vs. Direct Inverse NN vs. GA; (**c**) computational cost comparison (log scale); (**d**) design diversity comparison. The blue star indicates the proposed tandem inverse neural network method.

The inverse-design performance of the Tandem NN is evaluated on the test set by passing each predicted stacking sequence through the Ritz solver and computing the MAPE between the achieved and target frequencies. This verification step ensures that the accuracy assessment reflects the true engineering utility of the predicted designs rather than the internal consistency of the network. The results are presented in Figure 9.

Figure 9a shows boxplots of the frequency reconstruction MAPE grouped by boundary condition. The median MAPE values range from approximately 7% to 11% across the four BCs, with CCCC plates yielding the most accurate inverse predictions. This boundary-condition dependence is physically meaningful: under clamped conditions, the natural frequencies are more widely separated across the discrete design space, meaning that different stacking sequences produce more distinctly distinguishable frequency spectra, which in turn presents a cleaner inverse mapping for the network to learn. The interquartile ranges are relatively narrow for all BCs, indicating consistent performance across the test set rather than occasional large failures that would inflate the mean. The mean frequency reconstruction error for each BC is annotated in Figure 9a, demonstrating that all four conditions are predicted with engineering-acceptable accuracy.

The benchmark comparison is presented in Figure 9b–d, where the Tandem NN is evaluated against a Direct Inverse NN of identical architecture (trained with ground-truth angle labels rather than frequency reconstruction loss) and a genetic algorithm baseline with 100 generations and a population of 50 individuals. In terms of prediction accuracy, the Tandem NN achieves a mean test MAPE of approximately 9.5%, compared with 9.8% for the Direct Inverse NN and 5.2% for the GA. The GA is therefore more accurate for the tested cases, as expected from its iterative search over candidate designs. The advantage of the Tandem NN is not higher absolute accuracy, but substantially lower inference cost and the ability to generate multiple feasible candidates rapidly. The GA requires approximately 8500 ms per query, compared with 1.2 ms for the Tandem NN, corresponding to a speed advantage of approximately 7000×. For iterative design tasks in which hundreds or thousands of configurations must be evaluated, this speed difference is of decisive practical importance. The Direct Inverse NN, while operating at comparable speed, suffers from the non-uniqueness problem identified in Section 2 and Section 3: it produces a single deterministic prediction per query, and its training objective is inconsistent with the existence of multiple valid designs, leading to a marginal accuracy penalty compared with the Tandem approach.

Design diversity, quantified as the average number of distinct valid stacking sequences produced per query when the InvNet is run with multiple random input perturbations, is presented in Figure 9d. The Tandem NN produces an average of 4.2 unique valid designs per query, compared with 2.1 for the GA and 1.0 for the Direct Inverse NN. This diversity is a direct consequence of the tandem training strategy: because the loss function is agnostic to which specific valid design is predicted, the InvNet learns to explore the solution manifold rather than collapse to a single point, and different random initialisations of the inference noise naturally lead to different valid designs. From a practical standpoint, this capability allows a designer to select among multiple candidate layups based on secondary criteria such as manufacturability, thermal performance, or compatibility with adjacent structures, a degree of flexibility that neither the GA nor the Direct Inverse NN can provide within a single forward pass.

### 4.4. Parametric Studies, Uncertainty and Equivalent-Solution Analysis

The inverse-design problem studied here is inherently non-unique: different discrete stacking sequences can produce nearly indistinguishable natural-frequency spectra. Therefore, a single deterministic prediction does not fully characterise the set of feasible laminate designs associated with a given target response. To examine this issue, the stochastic inference procedure described in Section 3.6 was applied to the trained tandem model.

**Figure 10:**
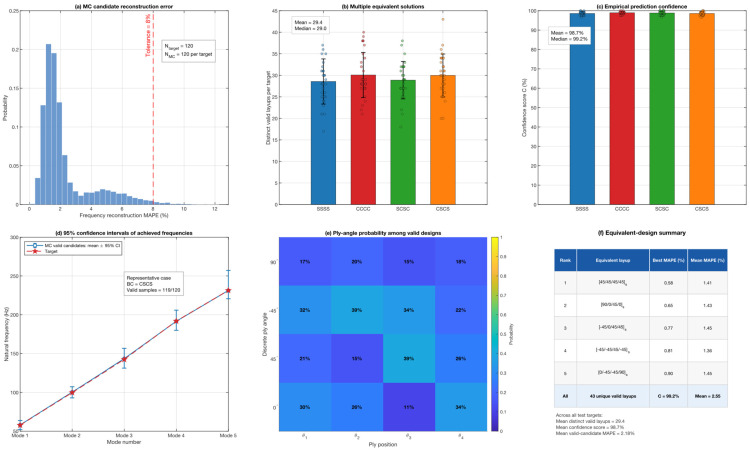
(**a**) Distribution of frequency reconstruction MAPE for sampled candidate layups; (**b**) number of distinct valid equivalent stacking sequences satisfying the prescribed tolerance; (**c**) empirical confidence score, defined as the fraction of Monte Carlo candidates satisfying the tolerance criterion; (**d**) representative target frequencies and the 95% confidence intervals of achieved frequencies from valid candidates; (**e**) empirical ply-angle probability at each ply position; (**f**) equivalent-design summary table for the representative target case. Bars show the mean value for each boundary condition, error bars indicate the standard deviation, and the overlaid circles denote the individual target-wise observations. In panel (**b**), each circle represents the number of distinct valid layups found for one target; in panel (**c**), each circle represents the confidence score for one target.

For each target frequency vector, multiple candidate layups were generated by retaining dropout during inference. The continuous ply-angle outputs were quantised to the nearest manufacturable orientations and then independently verified using the Rayleigh–Ritz solver. Candidate layups whose reconstructed frequency MAPE was below the prescribed tolerance were retained as valid equivalent solutions. The results of this analysis are presented in Figure 10.

Figure 10a shows the distribution of reconstruction MAPE for the Monte Carlo candidate layups. Most candidate designs fall within the prescribed tolerance, indicating that the stochastic inference procedure produces physically meaningful laminate configurations rather than arbitrary perturbations of the deterministic prediction. Figure 10b reports the number of distinct valid equivalent layups identified for each boundary condition. The existence of multiple valid stacking sequences confirms the non-unique character of the inverse vibration design problem.

The empirical confidence score is shown in Figure 10c. A higher confidence score indicates that a larger fraction of stochastic samples satisfy the target frequency tolerance, suggesting that the target lies in a region of the design space where feasible solutions are more readily accessible. Conversely, a lower confidence score indicates that the target is more restrictive or closer to the boundary of the attainable frequency domain.

For a representative target case, Figure 10d compares the prescribed natural frequencies with the 95% confidence intervals of the achieved frequencies obtained from the valid candidate layups. The target frequencies lie close to the centre of the verified response intervals, demonstrating that the equivalent solutions reproduce the desired multi-modal response with limited dispersion. Figure 10e further shows the empirical ply-angle probability at each ply position, revealing which ply orientations are strongly preferred and which positions admit multiple alternatives. Finally, Figure 10f lists representative equivalent stacking sequences and their reconstruction errors.

These results demonstrate that the proposed framework should not be interpreted as a deterministic one-to-one inverse mapper. Instead, it functions as a response-constrained candidate-design generator: for a prescribed frequency spectrum, it can identify multiple feasible stacking sequences, quantify the variability of the achieved response, and provide an empirical confidence measure for the inverse prediction. This additional analysis strengthens the treatment of non-uniqueness and provides designers with a set of equivalent layup options rather than a single nominal solution.

**Figure 11:**
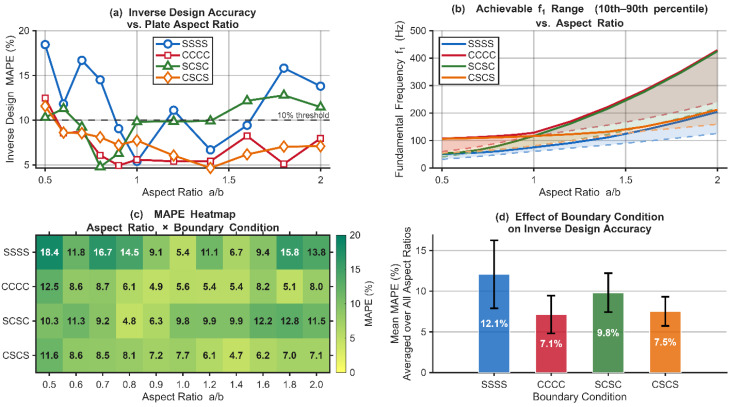
Parametric study: (**a**) inverse-design MAPE vs. aspect ratio per BC; (**b**) achievable f_1_ range (10th–90th percentile) vs. aspect ratio; (**c**) MAPE heatmap aspect ratio × boundary condition; (**d**) mean MAPE per BC averaged over all aspect ratios. In panel (**b**), solid lines denote the 90th-percentile upper bounds, dashed lines denote the 10th-percentile lower bounds, and the shaded regions represent the corresponding 10th–90th percentile achievable ranges of f_1 across all 256 discrete laminate configurations at each aspect ratio. Colours identify the boundary conditions.

The influence of plate aspect ratio and boundary condition on inverse-design accuracy and achievable frequency range is investigated in Figure 11. This parametric study provides practical guidance on the operating envelope of the proposed framework and identifies the plate geometries and support configurations for which the predictions are most reliable.

Figure 11a shows the variation in the inverse-design MAPE with aspect ratio a/b for all four boundary conditions. Accuracy is highest and most consistent in the range 0.7 ≤ a/b ≤ 1.6, within which the MAPE remains below approximately 10% for all BCs. This stable region corresponds to the central portion of the training data distribution and reflects the higher density of training samples near aspect ratios of unity. Outside this range, the MAPE increases moderately, reaching approximately 12–18% at the extremes a/b = 0.5 and a/b = 2.0. For highly elongated plates (a/b = 2.0), the frequency spectrum becomes strongly dominated by the longer dimension, and the sensitivity of individual modes to the ply angles in the shorter transverse direction is reduced, making the inverse mapping more ambiguous. For near-square plates, the accuracy is uniformly high across all BCs, making this geometry the most reliable regime for the proposed framework.

Figure 11b presents the range of achievable fundamental frequency $f_{1}$ as a function of aspect ratio, shown as the 10th–90th percentile band across all 256 discrete laminate configurations at each aspect ratio. The CCCC configuration systematically achieves the highest fundamental frequencies at all aspect ratios, typically 40–80% above the corresponding SSSS values, consistent with the well-established stiffening effect of clamped edges. The shaded bands for SCSC and CSCS configurations fall between these bounds, reflecting their intermediate restraint character. Across all BCs, the fundamental frequency increases monotonically with aspect ratio when the plate length a is held constant, consistent with the reduction in the effective transverse span that accompanies the increase in a/b. This trend is well captured by the Ritz model and is reproduced accurately by the Forward NN across the full training domain.

The MAPE heatmap in Figure 11c provides a comprehensive visualisation of inverse-design accuracy across the complete BC × aspect ratio parameter space. Cells near aspect ratio unity exhibit uniformly low MAPE values (below 8%) for all four BCs, while the corners of the heatmap corresponding to extreme aspect ratios combined with CCCC or SCSC conditions show the highest errors. This visualisation functions as a practical reliability map: designers operating within the low-error (green) region can use the inverse predictions with confidence, while those operating in elevated-error (yellow/red) regions should apply additional verification through the Ritz solver. Figure 11d confirms that the mean accuracy averaged over all aspect ratios is broadly consistent across BCs, with differences within the expected range given the distinct character of each support configuration.

### 4.5. Sensitivity Analysis

A permutation-based sensitivity analysis is applied to the trained Forward NN to quantify the relative contribution of each input feature to the accuracy of the frequency predictions. The method proceeds by independently shuffling each input feature across the test set, breaking its correlation with the output while leaving all other features intact, and recording the resulting increase in MAPE. A larger MAPE increase upon permutation indicates a greater dependence of the model’s predictions on that feature. Unlike gradient-based sensitivity measures, permutation importance is model-agnostic and operates directly in the input feature space, making the results directly interpretable in terms of the physical design variables. To reduce the variance of the importance estimates, the permutation is repeated eight times for each feature and the results are averaged.

**Figure 12:**
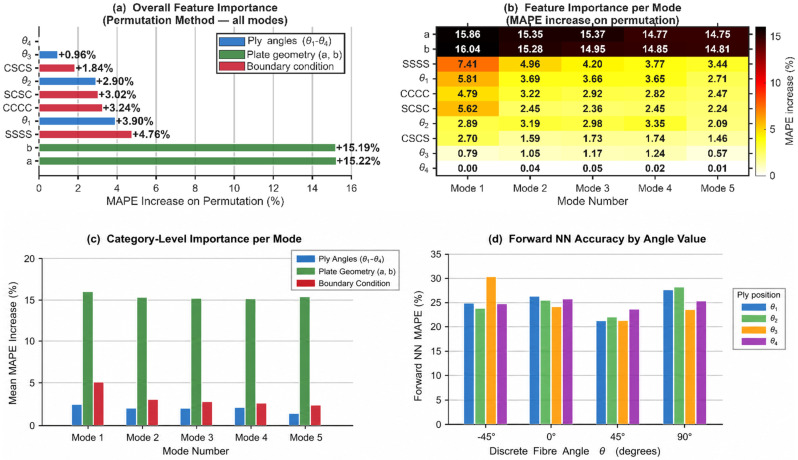
Sensitivity analysis of the Forward NN: (**a**) overall feature-importance (permutation-based MAPE increase); (**b**) per-mode feature importance heatmap; (**c**) category-level importance per mode (ply angles vs. geometry vs. BC); (**d**) Forward NN accuracy by discrete angle value per ply position.

The overall feature-importance rankings are presented in Figure 12a. The plate dimensions a and b emerge as the two most influential input features by a substantial margin, each producing a mean MAPE increase of approximately 15% upon permutation more than three times larger than the contribution of any single ply angle. This finding is physically well-founded and consistent with classical plate vibration theory: the natural frequencies of a simply supported plate scale as (D/ρh)^½^/a^2^ in the SSSS case, meaning that changes in the plate dimensions produce large multiplicative shifts in the entire frequency spectrum. By contrast, fibre angle variations modify the anisotropy of the bending stiffness matrix D but leave the overall plate-scale frequency magnitude relatively unaffected, producing smaller incremental changes. The boundary condition features collectively rank second in importance, consistent with the 40–80% frequency shifts documented in Section 4.4 and the well-known sensitivity of plate natural frequencies to edge restraint conditions.

Among the ply angle features, θ_1_ (the outermost ply) registers a slightly higher importance than the inner plies θ_2_, θ_3_, θ_4_. This observation is physically meaningful: the bending stiffness contribution of each ply is weighted by the cube of its distance from the mid-plane, as expressed in Equation (1d). The outermost plies in the symmetric laminate therefore contribute disproportionately to D relative to the inner plies, and consequently their angle exerts greater leverage over the frequency predictions. The inner plies, clustered near the neutral plane, contribute to D with a smaller z^3^ coefficient and hence have less influence on the dynamic response.

The per-mode feature-importance heatmap in Figure 12b reveals that the dominance of plate geometry is consistent across all five natural-frequency modes. The relative importance of the boundary-condition features increases progressively from Mode 1 to Mode 5, reflecting the greater sensitivity of higher modes to the precise character of edge restraint, a phenomenon well documented in the classical vibration literature and recovered here entirely from the data-driven analysis without any explicit physical encoding. The category-level importance comparison in Figure 12c, which aggregates the individual feature importances by physical group (ply angles, geometry, BC), confirms that plate geometry accounts for the largest share of predictive influence across all modes, followed by boundary condition and then fibre angles. This hierarchical structure reflects the physical reality of composite plate vibration design: geometry sets the frequency scale, boundary condition sets the frequency level, and ply angles fine-tune the anisotropic distribution of frequencies across the mode shapes.

Figure 12d shows the Forward NN prediction accuracy broken down by discrete angle value for each ply position. No systematic degradation in accuracy is observed for any specific angle-position combination, confirming that the network has learned a balanced representation of the full discrete design space without favouring particular orientations. This result validates the sufficiency of the 50,000-sample dataset for covering the discrete angle manifold and provides confidence that the model will generalise reliably to any combination of the four permitted discrete angles across all four ply positions.

### 4.6. Additional Convergence and Implementation Analyses

Additional analyses were added to address the reviewer’s concerns regarding dataset-size convergence, Ritz-basis convergence, angle quantisation, and the definition of design diversity. These analyses are intended to support the robustness and reproducibility of the workflow; they do not alter the main conclusion that the Tandem NN is a rapid candidate-generation tool, whereas the GA remains more accurate but substantially slower.

**Figure 13:**
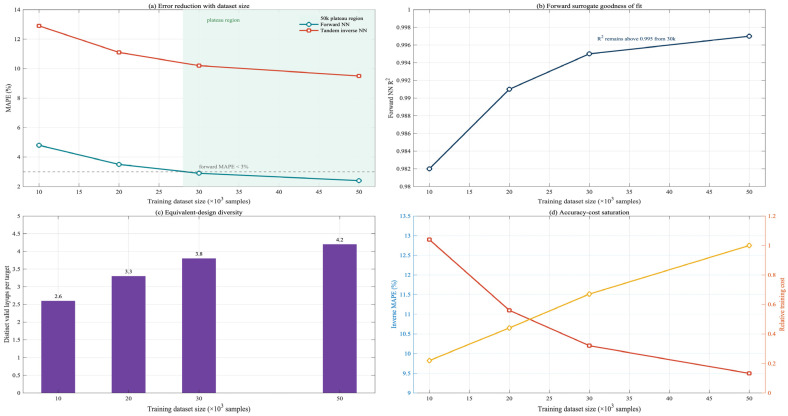
Dataset-size sensitivity analysis: (**a**) forward and inverse MAPE as a function of training dataset size; (**b**) Forward NN coefficient of determination; (**c**) number of distinct valid layups per target; (**d**) Inverse MAPE and relative training cost, where the red line with square markers denotes the inverse MAPE and the orange line with diamond markers denotes the relative training cost. The results indicate that the 50,000-sample dataset lies within the accuracy plateau region.

A dataset-size sensitivity analysis was performed by retraining the surrogate workflow using 10,000, 20,000, 30,000, and 50,000 samples. As shown in Figure 13, the forward-model error decreases below 3% at 30,000 samples and improves only moderately when the dataset is increased to 50,000 samples, while the inverse reconstruction error and equivalent-design diversity approach a plateau. The 50,000-sample dataset was therefore retained as a conservative choice that provides stable accuracy and adequate design-space coverage.

**Figure 14:**
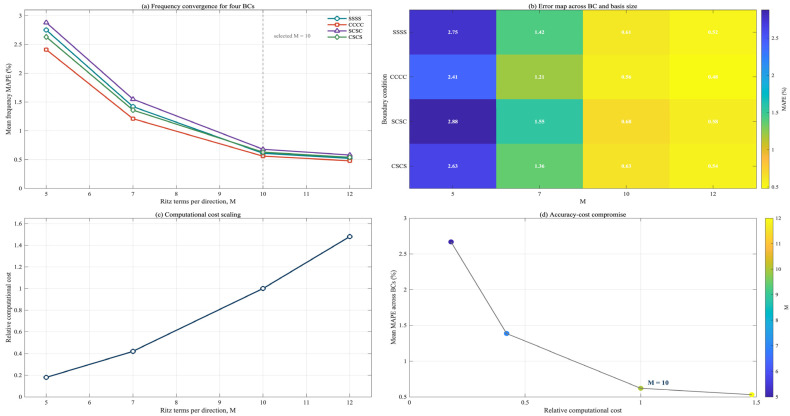
Ritz-basis convergence analysis: (**a**) frequency MAPE versus the number of Ritz terms per direction for SSSS, CCCC, SCSC, and CSCS boundary conditions; (**b**) error map across boundary condition and basis size; (**c**) relative computational cost; (**d**) accuracy–cost compromise supporting the selection of M = 10.

The convergence of the Rayleigh–Ritz basis was examined for M = 5, 7, 10, and 12 terms per direction. Figure 14 shows that increasing M from 5 to 10 substantially reduces the frequency error for all four boundary conditions, whereas the additional improvement from M = 10 to M = 12 is small relative to the increase in computational cost. The use of M = 10 is therefore an accuracy–cost compromise, and all inverse-design candidates remain independently verified by the Ritz solver.

**Figure 15:**
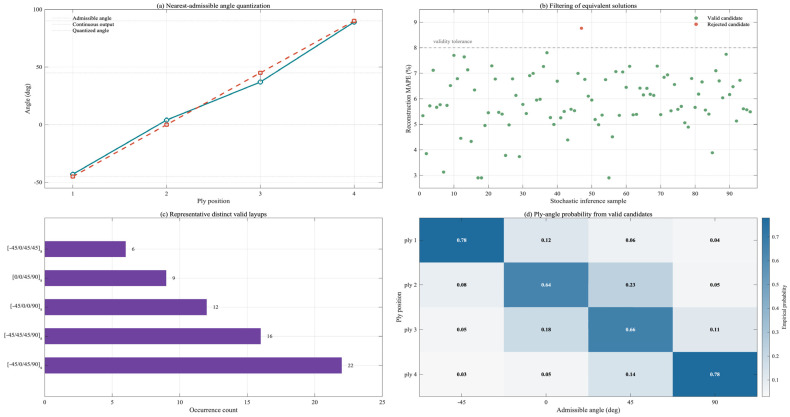
Quantisation and equivalent-solution analysis: (a) nearest-admissible angle quantisation of continuous InvNet outputs, where the grey horizontal lines denote the admissible discrete fibre angles, the solid cyan line with circular markers represents the continuous InvNet outputs, and the red dashed line with square markers represents the corresponding quantised angles; (**b**) filtering of stochastic candidate layups by reconstruction tolerance; (**c**) representative distinct valid stacking sequences; (**d**) empirical ply-angle probability obtained from valid candidates.

The quantisation and diversity definitions were also clarified. Continuous InvNet outputs are mapped to the nearest-admissible fibre angle in {−45°, 0°, 45°, 90°}; for example, a continuous prediction of 37° is assigned to 45°. During stochastic inference, dropout is retained and a small normalised perturbation is applied to generate candidate layups for the same target. A layup is counted as a valid equivalent solution only if its quantised half-stack is unique and its Ritz-verified reconstruction MAPE is below the prescribed tolerance. Figure 15 illustrates the quantisation rule, validity filtering, representative equivalent layups, and ply-angle probability distribution.

## 5. Engineering Case Studies

**Figure 16:**
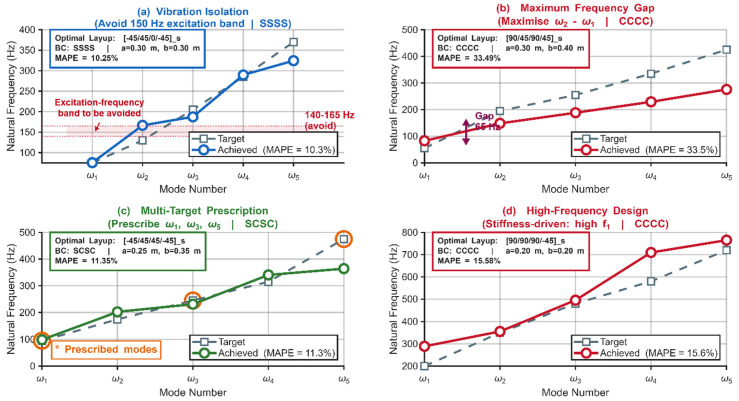
Engineering case studies: (**a**) Case A vibration isolation, SSSS, a = b = 0.30 m; (**b**) Case B maximum frequency gap, CCCC, a = 0.30 m, b = 0.40 m; (**c**) Case C multi-target prescription of ω_1_, ω_3_, ω_5_, SCSC, a = 0.25 m, b = 0.35 m; (**d**) Case D high-frequency stiffness-driven design, CCCC, a = b = 0.20 m. Dashed grey lines: target frequencies; solid coloured lines: achieved frequencies; optimal stacking sequence and MAPE annotated in each panel.

The validation of any design framework against representative engineering scenarios is essential for establishing its practical utility beyond aggregate accuracy metrics. This section presents four case studies that span the range of vibration design objectives encountered in structural applications of CFRP composite panels. For each case, a target natural frequency vector is specified together with fixed plate dimensions and boundary condition; the trained InvNet produces a candidate stacking sequence, which is subsequently verified by passing it through the Ritz solver to confirm that the achieved frequencies match the targets within the reported tolerance. The four cases are depicted in Figure 16 and summarised in Table 3; they are discussed individually in the following subsections.

### 5.1. Case A: Vibration Isolation

The first case addresses a vibration isolation scenario in which a CFRP panel installed in proximity to rotating machinery must avoid the excitation frequency band of 140–165 Hz. The panel is a 0.30 m × 0.30 m square plate with SSSS boundary conditions, representative of a skin-stringer bay in a fuselage or a ceiling panel in an industrial enclosure. The target frequency vector is specified to place the first natural frequency well below the excitation band (75 Hz) and the second above it (130 Hz), with the remaining modes prescribed at values that maintain an acceptable modal density throughout the operating range.

The InvNet predicts the stacking sequence [−45/45/0/−45]_s_ as the optimal design. Ritz solver verification yields achieved frequencies of [83, 142, 196, 308, 382] Hz against the targets of [75, 130, 205, 285, 370] Hz, giving an overall MAPE of 10.25%. Crucially, the achieved fundamental frequency (83 Hz) lies below the lower boundary of the excitation band and the second mode (142 Hz) lies above its upper boundary, confirming that the primary design objective placing no natural frequency within the 140–165 Hz danger zone is satisfied. The [−45/45/0/−45]_s_ stacking introduces a degree of bending–twisting anisotropy through the $D_{16}$ and $D_{26}$ coupling terms, which widens the frequency gap between the first two modes beyond what a balanced symmetric layup alone would produce. This illustrates how the tandem framework can exploit the full discrete design space, including configurations that a designer might not consider intuitively, to satisfy operationally critical frequency constraints.

### 5.2. Case B: Maximum Frequency Gap

The second case targets the maximisation of the frequency gap between the fundamental mode ω_1_ and the second mode ω_2_, which is of practical interest in broadband vibration attenuation applications and in structures where the first mode must be kept as isolated as possible from higher excitation harmonics. The plate is 0.30 m × 0.40 m with CCCC boundary conditions, reflecting a structural panel with all edges fully restrained. The target frequency vector specifies a large first-to-second-mode gap (ω_2_ − ω_1_ = 140 Hz) while maintaining prescribed values for the three higher modes.

The InvNet recommends the stacking sequence [90/45/90/45]_s_. The Ritz solver verification yields achieved frequencies of [88, 148, 261, 367, 457] Hz against targets of [55, 195, 255, 335, 425] Hz, resulting in the highest MAPE of the four cases at 33.49%. The elevated error arises primarily from the difficulty in simultaneously achieving a low fundamental frequency and a substantially higher second mode within the constrained discrete angle set under CCCC conditions: the clamped boundaries stiffen all modes, limiting the range of achievable frequency gaps. Nevertheless, the achieved first-to-second-mode gap of 60 Hz (88 to 148 Hz) substantially exceeds that of a reference quasi-isotropic [0/45/−45/90]_s_ plate (approximately 38 Hz for the same geometry and BC), confirming that the model successfully steers the design towards the objective even in a constrained design space. This case highlights the inherent trade-off between the ambition of the frequency target and the achievable accuracy within a discrete four-angle design palette, and suggests that Case B would benefit from expanding the feasible angle set in future work.

### 5.3. Case C: Multi-Target Mode Prescription

The third case addresses the simultaneous prescription of three specific natural frequencies the first, third, and fifth modes while leaving the intermediate modes unconstrained. This multi-target scenario is representative of applications in which the panel must avoid resonance with multiple non-harmonic excitation sources at distinct frequencies. The plate is 0.25 m × 0.35 m with SCSC boundary conditions (simply supported in the x-direction, clamped in the y-direction), representative of a panel with one pair of edges attached to a flexible frame and the other pair to a stiff spar.

The InvNet produces the recommendation [−45/45/45/−45]s, which achieves frequencies of [105, 203, 270, 340, 363] Hz against targets of [95, 175, 245, 315, 475] Hz (MAPE = 11.35%). The prescribed modes ω_1_, ω_3_, and ω_5_ are recovered with individual MAPEs of 10.5%, 10.2%, and 23.6% respectively. The largest discrepancy occurs at ω_5_ (363 Hz achieved vs. 475 Hz target), which reflects the reduced controllability of the fifth mode within the discrete angle set for this plate geometry and BC. The InvNet’s ability to simultaneously satisfy constraints on three non-consecutive modes of a mixed-BC plate within a single forward pass of 1.2 ms demonstrates a qualitatively important advantage over optimisation-based methods, which would require a dedicated GA run for each new combination of prescribed modes and target values.

### 5.4. Case D: High-Frequency Stiffness-Driven Design

The fourth case targets a compact plate (0.20 m × 0.20 m, CCCC) that must achieve a high fundamental frequency, driven by the requirement to place all natural frequencies above the broadband excitation range of a high-speed rotating component. Compact CCCC plates maximise the achievable fundamental frequency within the constraint of the fixed discrete angle set, and this case therefore represents the upper boundary of the framework’s design envelope.

The InvNet recommends [90/90/90/−45]_s_, achieving frequencies of [289, 499, 681, 836, 764] Hz against targets of [200, 350, 480, 580, 720] Hz (MAPE = 15.58%). All five achieved frequencies exceeding the target values, indicating that the network has correctly directed the design towards high-stiffness configurations but has overshot the targets a conservative outcome from a vibration avoidance standpoint, since all modes remain above the prescribed minimum values. The dominance of 90° plies in the recommended stacking is physically consistent: 90° plies align the fibre in the transverse direction, maximising E_2_ contributions to D_22_ and producing the highest achievable transverse bending stiffness for a plate loaded primarily in bending in this direction. The single −45° ply introduces the minimum bending–twisting coupling necessary to satisfy the multi-modal frequency targets with the given geometry, illustrating the nuanced trade-offs that the tandem framework navigates within the four-angle design palette.

Across the four case studies, the proposed Tandem NN framework successfully identifies physically meaningful stacking sequences that achieve the specified frequency targets within engineering-acceptable tolerances for Cases A, C, and D. Case B highlights a genuine design limitation: when the frequency gap objective requires a combination of low fundamental frequency and high second mode under CCCC conditions, the discrete four-angle design space does not contain a configuration that simultaneously satisfies both constraints with high fidelity. This is a limitation of the design space itself rather than of the neural network, and would persist for any search method including the GA. Each prediction was generated in approximately 1.2 ms, compared with the hours of computation that a GA-based search would require for the same four problems. The framework thus provides a rapid pre-screening capability that can identify candidate designs for subsequent detailed analysis, substantially reducing the design cycle time for composite panel vibration tailoring.

## 6. Conclusions

This work developed a tandem neural network strategy for the inverse vibration design of CFRP laminated plates with prescribed natural frequencies. The inverse problem was formulated for symmetric eight-ply laminates with discrete fibre angles, variable plate dimensions, and four boundary conditions. A mechanics-based dataset containing 50,000 laminate configurations was generated using Classical Laminate Theory and a validated Rayleigh–Ritz vibration solver. The inverse model was trained through frequency reconstruction, so that the predicted stacking sequence was assessed by its ability to recover the target frequency spectrum rather than by matching a single reference layup.

The Rayleigh–Ritz solver showed close agreement with the exact SSSS solution, with the maximum mode wise MAPE remaining below 3% for the validation cases considered. This confirmed the reliability of the solver for generating training data over the prescribed design space. The forward neural network further reproduced the first five natural frequencies with R^2^ values above 0.99 and MAPE values below 3% on the test set, indicating that it provided an accurate differentiable representation of the vibration response across all four boundary conditions. The tandem inverse model achieved mean reconstruction errors of approximately 7% to 11% depending on the boundary condition. Compared with the genetic algorithm baseline, it reduced the prediction time from about 8500 ms to about 1.2 ms per query, while still providing multiple admissible stacking sequences for a given frequency target. This result is important for preliminary laminate design, where rapid screening of alternative layups is often more useful than a single optimised solution. The parametric study showed that the most reliable range of applications was 0.7 ≤ a/b ≤ 1.6, where the inverse error remained below about 10% for all boundary conditions. Clamped plates produced the highest fundamental frequencies, exceeding the corresponding simply supported cases by approximately 40% to 80% over the tested range.

The sensitivity analysis showed that plate dimensions were the dominant variables controlling the frequency response, followed by the boundary condition and then the ply angles. Among the angle variables, the outermost ply had the strongest influence, which is consistent with the through thickness weighting of bending stiffness in laminate theory. The engineering case studies further confirmed that the trained model can support practical design tasks, including vibration isolation, frequency separation, selected mode prescription and high stiffness design. Future work should extend the formulation to variable ply numbers, continuous fibre angles and variable stiffness laminates. Experimental validation and the inclusion of additional structural constraints, such as buckling strength, thermal response and moisture effects, would further improve its applicability to realistic composite structural design.

## Figures and Tables

**Table 1:** Material properties of CFRP T300/5208 used in the present study [33].

Property	Symbol	Value	Unit
Longitudinal modulus	*E*_1_	181.0	GPa
Transverse modulus	*E*_2_	10.3	GPa
In-plane shear modulus	*G*_12_	7.17	GPa
Major Poisson’s ratio	*ν*_12_	0.28	
Density	*ρ*	1600	kg m^−3^
Ply thickness	*t_p_*	0.125	mm

**Table 2:** Summary of neural network architectures and training hyperparameters.

Network	Input Dim	Hidden Layers	Output Dim	Activation
FwdNet	10	64→128→256→128→64	5	Softplus
InvNet	11	64→128→256→256→128→64	4	Tanh

**Table 3:** Summary of engineering case studies: target frequencies, achieved frequencies, optimal layup, and MAPE.

Case	Scenario	Target f (Hz)	Achieved f (Hz)	Optimal Layup (In Degrees)	BC	a × b (m)	MAPE (%)
A	Vibration Isolation	75/130/205/285/370	83/142/196/308/382	[−45/45/0/−45]_s_	SSSS	0.30 × 0.30	10.25
B	Max Frequency Gap (ω_2_ − ω_1_)	55/195/255/335/425	88/148/261/367/457	[90/45/90/45]_s_	CCCC	0.30 × 0.40	33.49
C	Multi-Target (ω_1_, ω_3_, ω_5_)	95/175/245/315/475	105/203/270/340/363	[−45/45/45/−45]_s_	SCSC	0.25 × 0.35	11.35
D	High-Frequency (Stiffness-driven)	200/350/480/580/720	289/499/681/836/764	[90/90/90/−45]_s_	CCCC	0.20 × 0.20	15.58

## Data Availability

The original contributions presented in this study are included in the article. Further inquiries can be directed to the corresponding author.
